# Unlabeled data selection for active learning in image classification

**DOI:** 10.1038/s41598-023-50598-z

**Published:** 2024-01-03

**Authors:** Xiongquan Li, Xukang Wang, Xuhesheng Chen, Yao Lu, Hongpeng Fu, Ying Cheng Wu

**Affiliations:** 1 Faculty of Information Engineering and Automation, Kunming University of Science and Technology, Kunming, 650500 China; 2 Sage IT Consulting Group, Shanghai, China; 3 The University of North Carolina at Chapel Hill, Chapel Hill, USA; 4 University of Bristol, Bristol, UK; 5 Khoury College of Computer Sciences, Northeastern University, Seattle, USA; 6 University of Washington, Seattle, USA

**Keywords:** Computer science, Information technology, Software

## Abstract

Active Learning has emerged as a viable solution for addressing the challenge of labeling extensive amounts of data in data-intensive applications such as computer vision and neural machine translation. The main objective of Active Learning is to automatically identify a subset of unlabeled data samples for annotation. This identification process is based on an acquisition function that assesses the value of each sample for model training. In the context of computer vision, image classification is a crucial task that typically requires a substantial training dataset. This research paper introduces innovative selection methods within the Active Learning framework, aiming to identify informative images from unlabeled datasets while minimizing the number of required training data. The proposed methods, namely Similari-ty-based Selection, Prediction Probability-based Selection, and Competence-based Active Learning, have been extensively evaluated through experiments conducted on popular datasets like Cifar10 and Cifar100. The experimental results demonstrate that the proposed methods outperform random selection and conventional selection techniques. The superior performance of the novel selection methods underscores their effectiveness in enhancing the Active Learning process for image classification tasks.

## Introduction

Image classification technology has become pivotal across a range of sectors, including healthcare, public safety, and intelligent transportation. The development of a robust image classification model typically necessitates a substantial, well-labeled dataset. However, the availability of such datasets is often limited, and the process of labeling images can be labor-intensive and costly. This challenge significantly impedes the advancement and deployment of image classification models, especially in areas where rapid response is critical. The lack of scalability and the potential for reduced model performance due to a limited diversity in the labeled data impairs the model’s capacity to adapt to novel situations. Consequently, in environments where comprehensive datasets are scarce, strategically selecting a subset of unlabeled images for labeling becomes essential. This approach aims to construct a training set that is both efficient and effective, ensuring high accuracy in image classification tasks.

Active Learning represents a transformative approach in machine learning, focused on efficiently minimizing the burden and costs associated with labeling vast datasets. This technique strategically selects the most informative samples for labeling in each iteration, significantly reducing the size of the training set required to develop a high-performing model. This method is particularly beneficial in scenarios where data labeling is prohibitively expensive or labor-intensive. By employing Active Learning, we can not only develop robust image classification models under data-limited conditions but also adeptly adapt to dynamic data environments, such as those found in emerging social media platforms and high-resolution satellite imagery. The efficacy of Active Learning extends beyond image classification; it has been successfully implemented in diverse tasks, including image classification^1–3^, target detection^4^, and semantic segmentation^5,6^. This versatility underscores its immense potential for broader application across various domains within image classification and other areas of machine learning.

In the domain of Active Learning, various query strategies focus on selecting the most informative samples for current models. These strategies are typically divided into two categories: informativeness-based and representativeness-based. Informativeness-based methods prioritize unlabeled samples exhibiting the highest level of uncertainty, whereas representativeness-based methods emphasize sample diversity to mirror the underlying data distribution within the unlabeled data pool^7,8^. Despite their strengths, both approaches in Active Learning have faced criticism for over-focusing on data selection at the expense of considering the model’s capacity for comprehension. This oversight can lead to the selection of samples that exceed the model’s understanding, resulting in localized optimization and suboptimal performance. Furthermore, partitioning training data into excessive batches for training can markedly prolong the overall training duration. Consequently, there is a pressing need to devise more balanced and effective Active Learning methodologies. These should holistically encompass informativeness and representativeness, while also factoring in the model’s learning capabilities, to optimize both performance and efficiency in model training.

In response to the constraints inherent in conventional Active Learning strategies and driven by the necessity for efficient, adaptable, and high-performance image classification models, we introduce three innovative methods within the ambit of deep neural networks. These methods are conceptualized from a synthesis of existing literature, emphasizing the critical need to balance informativeness and representativeness in alignment with a model’s learning capacity.

Similarity-based Selection: This method evaluates the resemblance between unlabeled and labeled image datasets. It ensures that the selected unlabeled data accurately represent the already labeled dataset, mitigating the selection bias often encountered in uncertainty-based selection methods. This approach enhances the coverage of the data distribution space, ensuring a more comprehensive representation.

Prediction Probability-based Selection: Central to this method is the evaluation of the initial deep learning model’s classification performance on unlabeled datasets. It involves assessing the probability of each unlabeled data belonging to specific categories, thereby informing the subsequent training cycle. By anchoring the selection on the model’s current performance with the unlabeled data, we ensure that the newly integrated data is both informative and within the model’s learning capability.

Competence-based Active Learning: Drawing inspiration from human pedagogical techniques, which typically progress from simpler to more complex concepts, this method tailors the selection strategy to match the model’s learning progression and capacity. This is particularly crucial for deep learning models, which are susceptible to stagnation in local optima.

Our contributions are threefold: Firstly, we address the need for a balanced approach in Active Learning that aligns data selection with the model’s evolving learning capacity. Secondly, our proposed methods collectively facilitate a more effective and efficient training process, catering to the dynamic requirements of image classification tasks. Thirdly, Our experimental studies on the Cifar10 and Cifar100 datasets^9^ demonstrate that our innovative Active Learning methods surpass existing techniques in both efficacy and stability within the realm of image classification tasks. Key findings reveal that our approaches not only elevate the model’s generalization capacity but also significantly curtail the training duration compared to prior methodologies. By pioneering these novel solutions, our research aims to close prevailing gaps and catalyze progress in the field of image classification technology.

## Related work

The field of Active Learning, particularly in image classification, has witnessed substantial advancements and diverse methodological approaches. This section provides an overview of these developments, categorizing them into key thematic areas and elucidating their respective contributions and limitations.

### Active learning

Active Learning has proven to be effective in various applications, including image classification^1,3,10,11^, image retrieval^12^, image captioning^13^, object detection^14^, and regression^15,16^. In recent years, Active Learning strategies have been categorized into three main categories: informativeness^16–20^, representativeness^8,10^, and hybrid approaches^21,22^. Informativeness-based methods focus on selecting the most uncertain samples to clarify the areas of highest uncertainty in the model’s knowledge, thus promoting more robust learning. Representativeness-based methods aim to enhance the model’s generalization ability by exposing it to diverse training examples, ensuring the selected samples capture the full range of data variability. Hybrid approaches combine both informativeness and representativeness criteria to extract the most valuable information from the unlabeled data pool, thereby optimizing the efficiency and effectiveness of the learning process. Despite the success of these strategies, each method has its limitations. Informativeness-based methods, while focusing on selecting samples with the highest uncertainty, often overlook that not all diverse samples are equally beneficial for training. Representativeness-based methods enhance the model’s generalization ability by exposing it to diverse examples but might miss informative yet rare instances. Hybrid approaches, though aiming to balance these two criteria, often introduce increased complexity and computational costs. Our work aims to develop novel active learning strategies that address these limitations, maintaining high performance in image classification tasks.

### Informativeness-based method

Informativeness-based approaches are considered as the best strategy in Active Learning and they can be categorized into bayesian^16^ and non-bayesian^23^ frameworks. The bayesian approach, such as the one proposed by Gal et al.^16^, uses Monte Carlo Dropout to estimate the uncertainty of the unlabeled data. However, this method requires dense dropout layers, which can reduce the convergence speed and result in huge computational costs for large-scale learning. In contrast, non-Bayesian frameworks like the one proposed by Li et al.^23^ utilize an information density measure, defined as a metric to evaluate the significance of an unlabeled data sample by its proximity to other samples in the feature space, along with an uncertainty measure, to select pivotal instances for labeling. Non-Bayesian frameworks, using information density and uncertainty measures, select pivotal instances for labeling but may exhibit bias toward densely populated data regions, potentially overlooking valuable outliers. Recent work has attempted to overcome these limitations, for instance Ash et al.^24^ proposed a new method to measure data uncertainty by calculating the expected gradient length, while Li et al.^25^ rethought the structure of the loss prediction module and defined the acquisition function as a learning-to-rank problem. Yoo et al.^7^ and He et al.^26^ employ a loss module to learn the loss of a target model and select data based on their output loss. Despite these advancements, the challenge of balancing model uncertainty with computational efficiency and holistic data representation still persists. Overemphasis on uncertainty often leads to biases towards more challenging samples, leaving out simpler yet diverse instances that could improve the model’s overall generalization capability. Our methods, such as Prediction Probability-based Selection, are developed to address these biases by considering a balance between data density and informativeness, thus enhancing the Active Learning process.

### Representativeness-based method

Representativeness-Based method usually uses a pre-trained self-supervised model to cluster the unlabeled data and select samples from each cluster that are most dissimilar to the already labeled samples. This method achieves good results in image classification tasks while reducing the number of labeled samples needed for training. In summary, representativeness-based methods focus on selecting diverse samples to enhance the robustness and generalization ability of the model. Sener et al.^8^ regard the step of selecting data as a issue of finding a current optimal set. In other words, a fixed number of samples are selected from the unlabeled data for the sake of adding to the set, and the newly added samples need to satisfy the maximum Euclidean distance from the samples in the set. The Core set method has been proved to be a relatively successful Active Learning algorithm. The Core-set method single out the samples by minimizing the Euclidian distance between the unlabeled data and labeled data in the feature space. However, the performance of this method is critically restricted by the data category in the datasets. To address this, Sinha et al.^27^ instead employ an adversarial approach to diversity-based sample query, which selects the unlabeled data based on the discriminator’s output, regards it as a selection criteria. However, the effectiveness and robustness of this approach can be influenced by the quality and reliability of the discriminator’s output. Furthermore, Bengar et al.^28^ integrated Active Learning with self-supervised pre-training, but further evaluation and analysis are required to assess its specific implementation and performance in different scenarios. While effective in enhancing the model’s robustness, they face limitations in accurately selecting the most informative and representative samples, impacting the efficiency of the sample selection process. These limitations include potential biases in diversity selection, the influence of discriminator output quality that refers to the effectiveness of a discriminator model, typically used in adversarial learning, in distinguishing between different categories or classes of data. Therefore, there is a need for further advancements and novel approaches to address these limitations and improve the overall sample selection process in Active Learning. Our Competence-based Active Learning method addresses the limitations of these representativeness-based methods by integrating the model’s learning capacity with the selection process, thus ensuring a more effective and balanced Active Learning approach.

### Curriculum learning

The concept of Curriculum Learning draws inspiration from the learning process observed in humans and animals, where they gradually take on more challenging tasks once they have mastered easier ones. This approach has been a topic of interest for many years^29^. In traditional machine learning, models often randomly select small batches of data from the training set and update the model parameters using stochastic gradient descent. In machine learning models, this approach can prevent models, especially deep learning models, from getting trapped in local optima, leading to better generalization performance. However, the random selection of data in traditional models can result in prolonged training times and subpar performance.

In response to the insights from the study in reference^30^, we have innovated a novel method for data selection in the realm of image classification. This technique focuses on evaluating the complexity of unlabeled images relative to the model’s current learning trajectory, using the model’s own probability estimates for its predictions as a basis. Our methodology is an intersection of Curriculum Learning principles and Active Learning techniques, crafted to progressively introduce more challenging samples to the model, thereby fostering a more robust learning process. We have refined this approach with the incorporation of a competence-based strategy, which calibrates the complexity of training samples to the model’s evolving learning stage. Rigorous experimentation has established that our method not only significantly reduces the number of training iterations required but also markedly enhances the model’s generalization capabilities.

## Method

**Figure 1:**
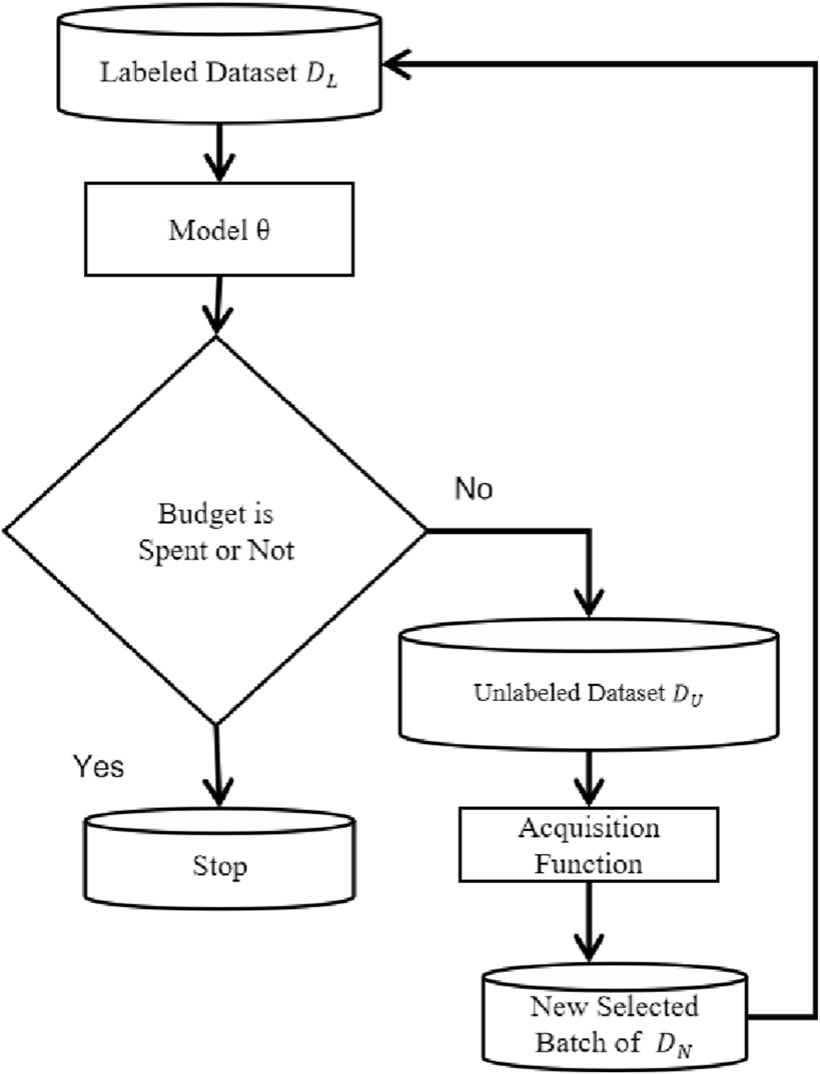
An overview of our active learning framework.

Our image classification framework is based on Active Learning, which involves a large pool of unlabeled data $D_{U}$ and a labeled dataset $D_{L}$. In each cycle, we select N samples for annotation to maximize our classification model’s performance. Active Learning methods typically allocate the budget sequentially over a few cycles, with the batch-mode variant labeling b samples per cycle as the only viable option for CNN^31^ training. At the start of each cycle, we train the model on the labeled set $D_{L}$ and then use our proposed acquisition function to select a certain amount of data from $D_{U}$ to add to $D_{L}$. We then retrain the model using the updated dataset and repeat this process until all selected samples are trained. The acquisition function is the most crucial component and the main point of difference among Active Learning methods in the literature. Figure 1 provides an overview of our Active learning framework, our objective is to identify the most valuable images from unlabeled datasets, which can enhance the model’s performance. To achieve this goal, we have developed methods for selecting informative images for image classification within an Active Learning framework.

In our research, we have developed two distinct data selection methodologies: Similarity-based Selection, dedicated exclusively to data selection, and Prediction Probability-Based Selection, which is integrated with our novel Competence-based Active Learning framework for model training. Our approach to data selection is twofold: one aspect focuses on the inherent characteristics of the data, while the other considers the requirements of the model in use. From the data’s standpoint, we prioritize the diversity and richness of information encapsulated in the images during our selection process. In terms of model considerations, we strategically select data that provides the most value to the model’s current stage of learning. Specifically, in the domain of image classification, our selection criterion hinges on assessing the difficulty level of classifying each image, tailored to the model’s current capabilities.

### Similarity-based selection

We use image embeddings to calculate the similarity between images and exclude images that are similar to those already selected. Image embedding is a low-dimensional continuous image space representation. To estimate the score of an image, we follow these steps:

1 $$Sscore\left(s\right)=\underset{l\in {D}_{L}}{\mathit{max}} \left(cos\left(emb\left(s\right),emb\left(l\right)\right)\right)$$

**Figure 2:**
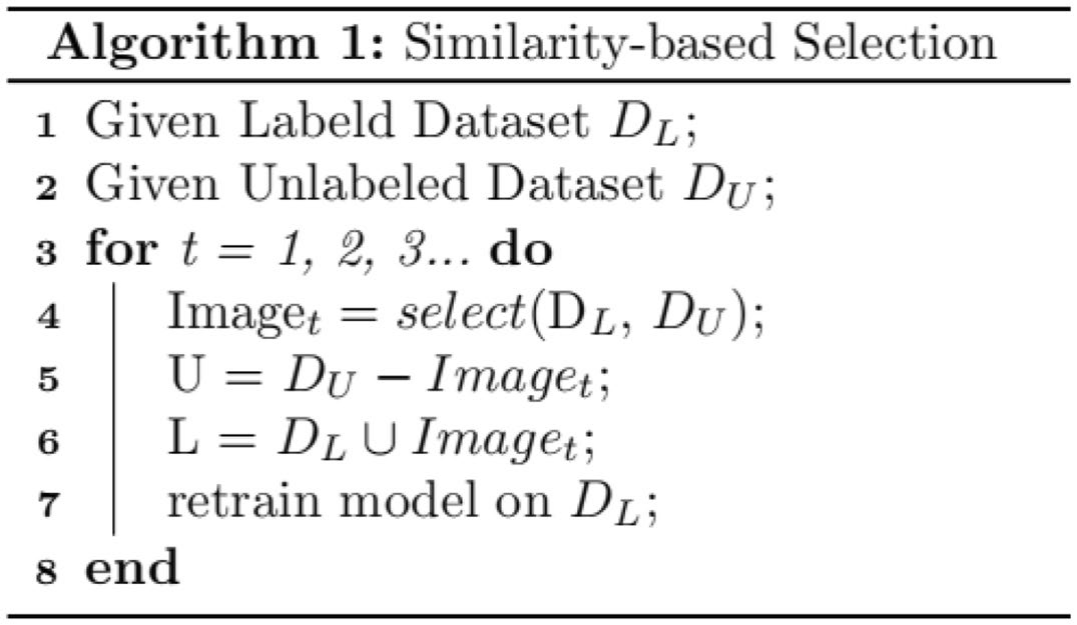
The algorithm 1.

The score of an image s is determined using its embedding, represented by emb(s). To compute this score, we first calculate the similarity between image s and all the images in the labeled datasets. The largest value of this similarity is selected as the similarity of image s to the labeled datasets. A higher $Sscore(s)$ indicates that image s is more dissimilar to the labeled datasets. We select image s to be included in $D_{L}$, if it has significant image information and is not redundant with high probability. We believe that these dissimilar images are more valuable to the current model than other similar images, considering the image information. At each iteration, we select the top N images based on their $Sscore(s)$ and add them to $D_{L}$ while removing them from $D_{U}$. The Fig. 2 shows the algorithm.

### Prediction probability-based selection

To enhance the selection of images for Active Learning, we propose using the Prediction Probabilities of images in image classification. Firstly, we train an Image Classification Model using labeled images. We then use this model to predict the labels of images in $D_{U}$ that are not labeled. Some images are predicted with high accuracy while others are not. We assume that the images with poor predictions contain new information that the current model needs to learn, while the images with high accuracy have already been learned. Based on this assumption, we select the images with poor predictions to be added to the labeled datasets, which helps to strengthen the model’s learning ability.

When using a pre-trained model to predict unlabeled images, we can compute the probability of each category to which the samples may belong. This is done by obtaining the output probabilities from the softmax layer of the model.

2 $$Probset\left({D}_{U}^{i}\right)=\left\{Prob\left({D}_{U}^{i}\in {c}_{0}\right),Prob\left({D}_{U}^{i}\in {c}_{1}\right),\cdots \cdots ,Prob\left({D}_{U}^{i}\in {c}_{n}\right)\right\}$$

In general, we select the category with the highest probability for image classification. If the maximum probability has a significant advantage over the probabilities of other categories, we consider the model to be confident in its prediction. However, if the maximum predicted probability is close to the probabilities of other categories, the model may have difficulty accurately classifying the sample. Therefore, we use the prediction probability as a score to select images for Active Learning. If the maximum predicted probability is high, we assume that the model has learned the information in the sample and does not need to be added to the labeled datasets. If the maximum predicted probability is not high enough, we assume that the model needs to learn the information contained in the sample, and thus the sample should be added to the labeled datasets.

3 $$PScore\left(i\right)=\frac{1-max\left(Probset\left({D}_{U}^{i}\right)\right)}{max\left(Probset\left({D}_{U}^{i}\right)\right)}$$

**Figure 3:**
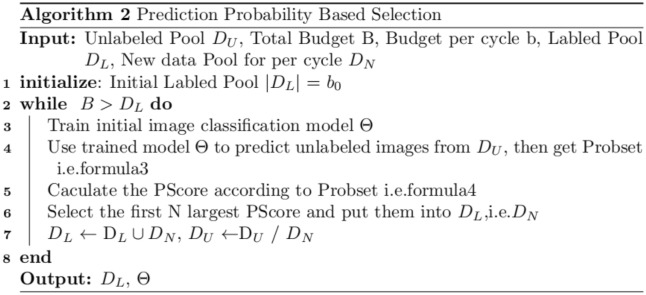
The algorithm 2.

The larger the $PScore(i)$ is, the worse the sample is predicted. Hence we select the first N largest $PScore(i)$ images as the new samples and add them to $D_{L}$ meanwhile remove the new samples from $D_{U}$. Algorithm 2 as follows (Fig. 3).

### Competence-based active learning

We have proposed a training framework called Competence-Based Active Learning, which combines Prediction Probability-Based Selection with curriculum learning^32^. The idea behind this framework is that by selecting training samples that are appropriate for the current level of competence of the model, we can improve its performance. We define two central concepts for this framework, as follows:

*Difficulty:* The learning difficulty of a sample can be defined as its $PScore(i)$ , which indicates how poorly the sample is predicted by the current model. When an unlabeled sample has a low $PScore(i)$ , it suggests that the current model struggles to predict it accurately, indicating that the sample is difficult to classify. Therefore, we can use $PScore(i)$ as a measure of the difficulty of a sample for the current model.

4 $$Difficulty\left(i\right)=PScore\left(i\right)$$

*Competence:* The competence of a learner can be seen as a value between 0 and 1, which reflects the learner’s growth during its training. This value is defined as a function of the learner’s state, specifically as the proportion of unlabeled data that the learner is allowed to select from at a given time t (measured in terms of training steps). The unlabeled data are ranked based on their difficulty, and the learner is allowed to use the top $c(t)$ portion of them at time t.

**Figure 4:**
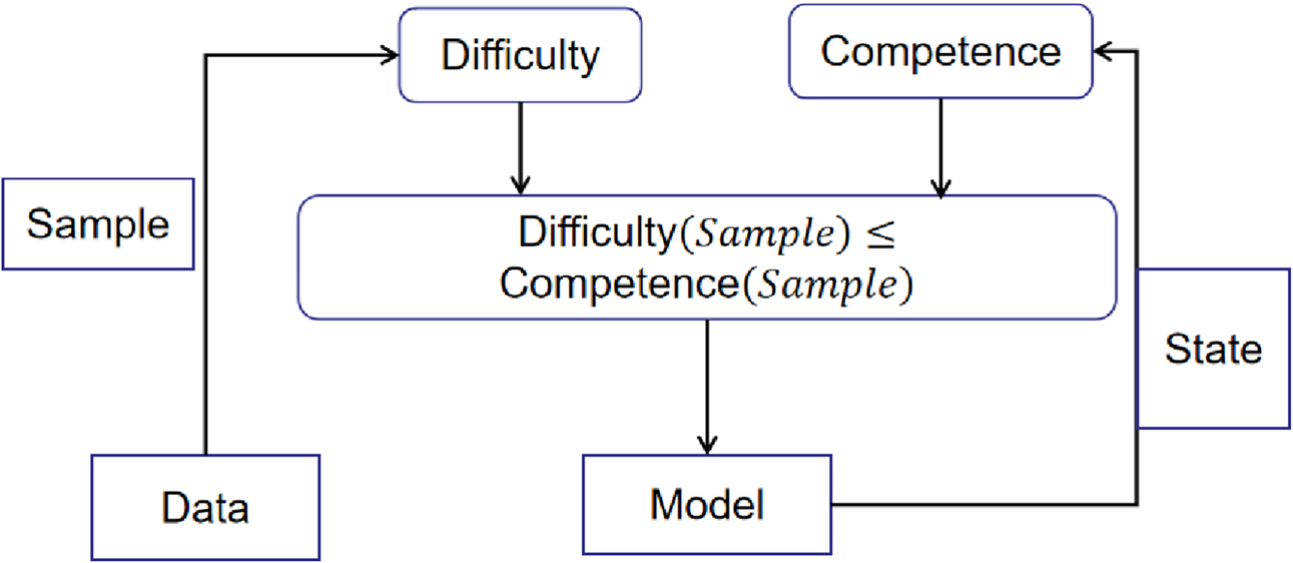
Curriculum learning.

In the condition of the root form, compared to other functions, $c(t)$ can obtain best performance to measure the competence of current model, because of that in our paper we define $c(t)$ as follows:

5 $${C}_{sqrt}\left(t\right)\triangleq min\left(1,\sqrt{t\frac{1-{C}_{0}^{2}}{T}+{C}_{0}^{2}}\right)$$

where $T$ is the total duration of the curriculum learning stage and $C_{0} \triangleq C(0) \ge 0$. An example of the relation between difficulty and competence is shown in Fig. 4. We also present Algorithm 3.

**Figure 5:**
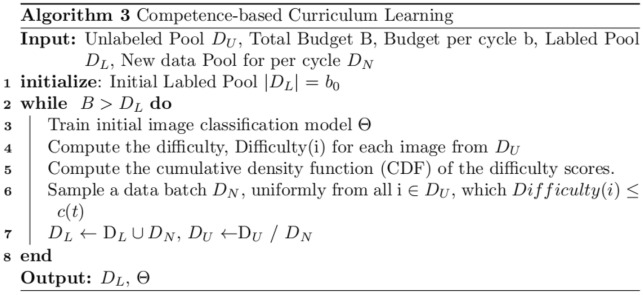
The algorithm 3.

As shown in Fig. 5, our proposed method for Competence-Based Active Learning consists of a systematic approach to selecting training samples based on the estimated difficulty of a sample and the current competence of the model. At each training cycle, we calculate the model’s competence value, which ranges from 0 to 1. If the competence value is greater than the difficulty value, then we select images to add to the labeled datasets for the next training cycle. This process is repeated until the model reaches a satisfactory level of performance.

## Experiment

### Datasets

CIFAR-10 and CIFAR-100 are widely used benchmark datasets in the field of computer vision. The CIFAR-10 datasets contain 50,000 training images and 10,000 test images, with each image belonging to one of 10 classes: airplane, automobile, bird, cat, deer, dog, frog, horse, ship, and truck. The CIFAR-100 datasets also contain 50,000 training images and 10,000 test images, but with each image belonging to one of 100 fine-grained classes, which are grouped into 20 coarse-grained classes.

Both datasets have an image size of 32 × 32 pixels and the images are in RGB color format. The CIFAR-10 and CIFAR-100 datasets are challenging benchmark datasets for image classification tasks and have been widely used in many research studies in the field of computer vision.

### Experimental setup

For our experiments, we randomly selected 10% of the training set as our initial labeled set, which consisted of 5000 images. The remaining 45,000 images were used as the unlabeled set. Our implementation of the proposed method is based on the PyTorch deep learning framework^33^. We used a pre-trained ResNet-18^34^ model as our base model for all experiments.

We implemented the Competence-Based Active Learning method as described in the previous sections. We used Prediction Probability Based Selection to rank the unlabeled images in terms of difficulty, and used the competence function to decide how many of the top difficult images to add to the labeled set at each iteration. We trained the model using stochastic gradient descent (SGD) with a learning rate of 0.1, momentum of 0.9, and weight decay of 5e−4. We train the models for 100 epochs and reduce the learning rate by a factor of 0.5 at 60 and 80 epochs. During the training process, we apply standard data augmentation techniques, such as random cropping from zero-padded images, random horizontal flipping, and image normalization by the channel mean and standard deviation computed over the training set. To efficiently solve the optimization problem, we use the Python scikit-image library. The reported results are averaged over 3 runs, and the evaluation metric is the accuracy on the test set.

We performed experiments with different sizes of the initial labeled set and different values for the competence function. We also compared our method to several baseline methods, including Random Sampling, Uncertainty Sampling, and Query by Committee. We evaluated the performance of the models based on their accuracy on the test set after each iteration.

### Baselines

In our study, we conducted comprehensive comparisons of our proposed methods with a range of baseline methods: Random Sampling, Uncertainty Sampling, Query by Committee (QBC), BALD (Bayesian Active Learning by Disagreement), EMC (Expected Model Change), and Bayesian Generative AL. The rationale behind selecting each of these baseline methods for comparison is as follows:

*Random Sampling:* As a fundamental benchmark in Active Learning, Random Sampling involves selecting data samples randomly without any strategic criteria. This baseline is crucial for demonstrating the effectiveness of our methods compared to a non-strategic approach.

*Uncertainty Sampling:* A prevalent strategy in Active Learning, Uncertainty Sampling selects samples based on the uncertainty of the model’s predictions. It serves as an important comparison point to showcase the improvements our methods offer over traditional Active Learning approaches, particularly in balancing informativeness and representativeness.

*Query by Committee (QBC):* QBC involves multiple models to select samples upon which the committee members most disagree. This baseline provides a robust comparison to our methods, allowing us to demonstrate their effectiveness against a collaborative, consensus-based approach.

*BALD (Bayesian Active Learning by Disagreement):* BALD utilizes Bayesian principles to select samples based on the model’s prediction disagreement. This method represents a sophisticated Active Learning strategy that combines uncertainty and ensemble techniques.

*EMC (Expected Model Change):* EMC selects samples based on the expected change in the model’s output. It is a forward-looking approach that prioritizes samples anticipated to have the greatest impact on the model’s future learning trajectory.

*Bayesian Generative AL:* This method employs Bayesian generative models for sample selection, focusing on the probabilistic modeling of data. It is particularly effective in scenarios where understanding the underlying data generation process is crucial for sample selection.

The comparison with these baseline methods was conducted on CIFAR10, CIFAR100, MNIST, and ImageNet datasets to evaluate the performance of our methods in terms of effectiveness and stability in image classification tasks, providing a holistic view of their strengths and weaknesses in various contexts.

### Experimental results

#### Performance on CIFAR10

**Figure 6:**
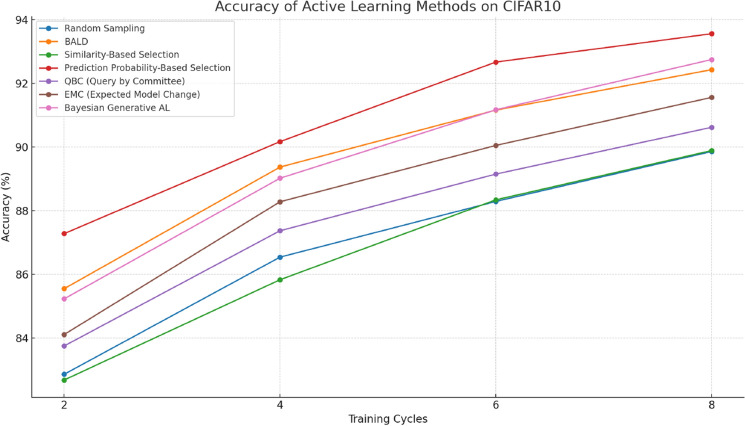
Accuracy of comparison methods on CIFAR10.

The accuracy metrics presented in this study refer to top-1 accuracy, which measures the percentage of times the model’s highest-probability prediction matches the true label. Tables 1 and 2 provide clear evidence of the varying performances of different methods. In particular, Fig. 6 demonstrates that similarity-based and prediction probability-based selections outperform random sampling in CIFAR10. However, the performance of similarity-based selection is notably volatile, while random sampling outperforms similarity-based selection in the initial few rounds of training. After eight rounds of training, similarity-based selection achieves a 0.03% higher accuracy than random sampling. Overall, if sufficient training rounds are completed, similarity-based selection is superior.

**Table 1:** Accuracy of active learning methods on CIFAR10.

Method	Cycle 2 (%)	Cycle 4 (%)	Cycle 6 (%)	Cycle 8
Random sampling	82.86	86.54	88.29	89.86%
BALD	85.55	89.37	91.16	92.43
Similarity-based selection	82.68	85.83	88.34	89.89
Prediction probability-based selection	87.28	90.17	92.67	93.56
QBC (query by committee)	83.75	87.37	89.15	90.62
EMC(expected model change)	84.11	88.28	90.05	91.56
Bayesian generative AL	85.23	89.02	91.17	92.75

**Table 2:** Accuracy of competence-based active learning on CIFAR10.

Cycles	Cycle 2	Cycle 4	Cycle 6	Cycle 8
Number of labeled data	8181	13,059	21,516	25,000
Competence-based active learning	81.02%	87.32%	92.95%	92.25%

In each cycle of training, prediction probability-based selection achieves approximately 3% higher accuracy than random sampling. Moreover, at certain training cycles, the accuracy of prediction probability-based selection exceeds that of BALD, and after eight rounds of training, its accuracy is 1.13% higher than BALD. It should be noted that competence-based active learning yields a high accuracy of 92.25% with just four rounds of training, reducing the time required for training and achieving higher accuracy than previous methods. To provide a comprehensive comparison, we included additional Active Learning methods, namely QBC (Query by Committee), EMC (Expected Model Change), and Bayesian Generative AL. The results, as shown in Table 1, indicate that Bayesian Generative AL achieves the highest accuracy, reaching 92.75% by the eighth cycle. This method’s performance is particularly notable, as it consistently outperforms QBC and EMC, demonstrating its efficacy in the CIFAR10 dataset context.

#### Performance on CIFAR100

**Figure 7:**
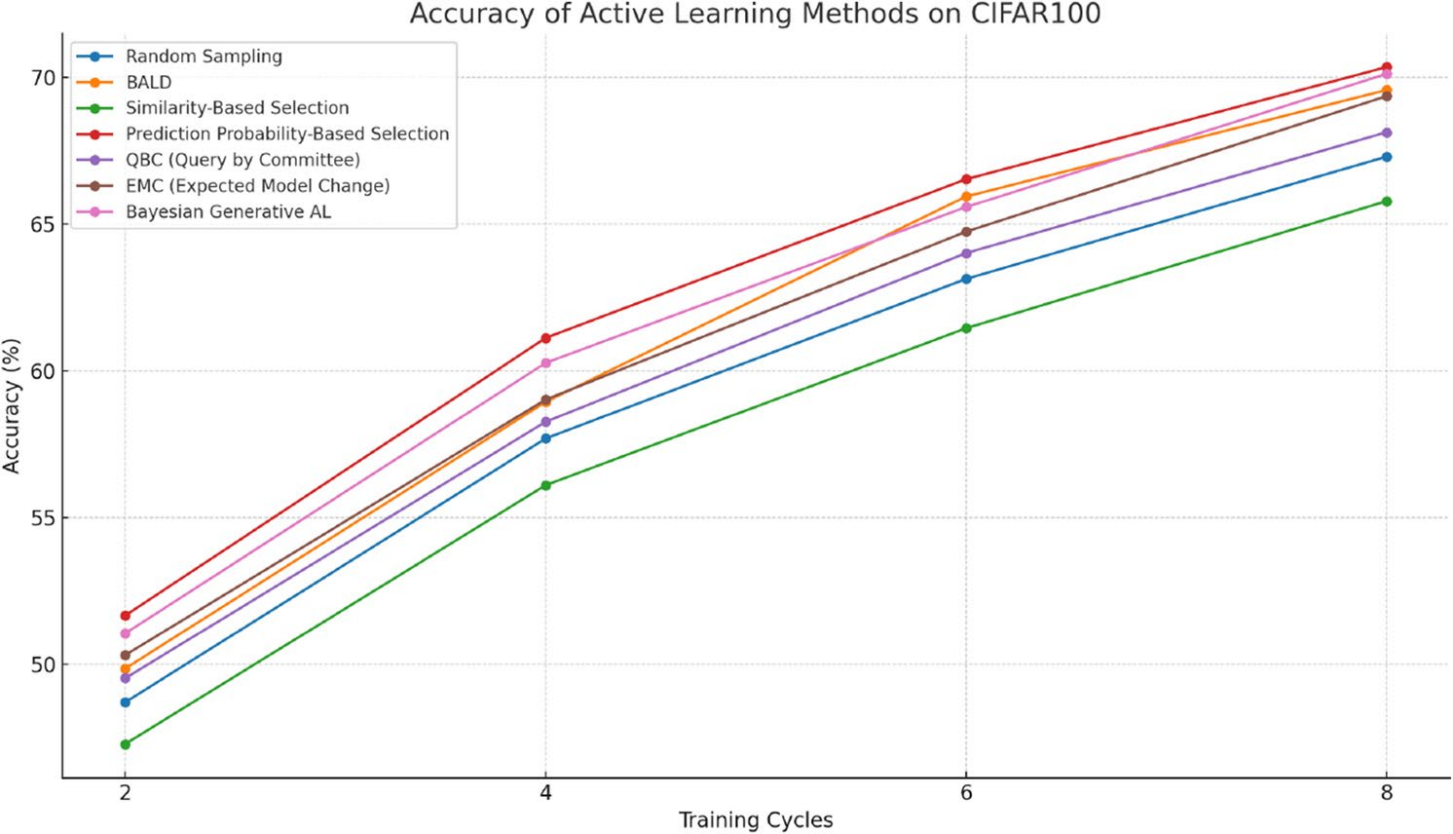
Accuracy of comparison methods on CIFAR100.

Tables 3 and 4 show clear differences in performance between the different active learning methods on CIFAR100. Similarity-based selection performs worse than Random sampling at each round, with lower accuracy. As shown in Fig. 7, Prediction Probability based selection achieves good performance, but during the first three rounds, its accuracy improvement over Random sampling is not significant. However, there is a significant improvement in accuracy starting from the fourth round. Active Learning iterates for 8 rounds before ending, but achieves a slightly higher final accuracy than Prediction Probability based selections. Table 3 quantitatively reflects the performance differences between the different methods, with Similarity-based selections having a final round accuracy 1.52% lower than Random sampling. Prediction Probability based selection achieves an average improvement of 3.21% over Random sampling and 1.41% over BALD. Similarly, on the CIFAR100 dataset, we evaluated the same set of additional Active Learning methods. As per Table 3, Bayesian Generative AL again shows superior performance, achieving an accuracy of 70.13% in the eighth cycle. This method surpasses the other tested methods, including QBC and EMC, across all training cycles, affirming its robustness and effectiveness on a more complex dataset like CIFAR100. Competence-based Active Learning achieves a growing improvement of 4.91% on average over Prediction Probability based selections across the cycles.

**Table 3:** Accuracy of active learning methods on CIFAR100.

Method	Cycle 2 (%)	Cycle 4 (%)	Cycle 6 (%)	Cycle 8
(%) Random sampling	48.71	57.70	63.14	67.31
BALD	49.86	58.95	65.95	69.58
Similarity-based selection	47.29	56.11	61.46	65.79
Prediction probability-based selection	51.67	61.13	66.54	70.36
QBC (Query by Committee)	49.53	58.27	64.02	68.14
EMC(Expected Model Change)	50.32	59.03	64.75	69.37
Bayesian generative AL	51.06	60.28	65.59	70.13

**Table 4:** Accuracy of competence-based active learning on CIFAR100.

Cycles	Cycle 2	Cycle 4	Cycle 6	Cycle 8
Number of labeled data	18,514	21,902	24,801	25,000
Competence-based active learning	64.23%	66.32%	67.56%	71.14%

#### Performance on MNIST

**Figure 8:**
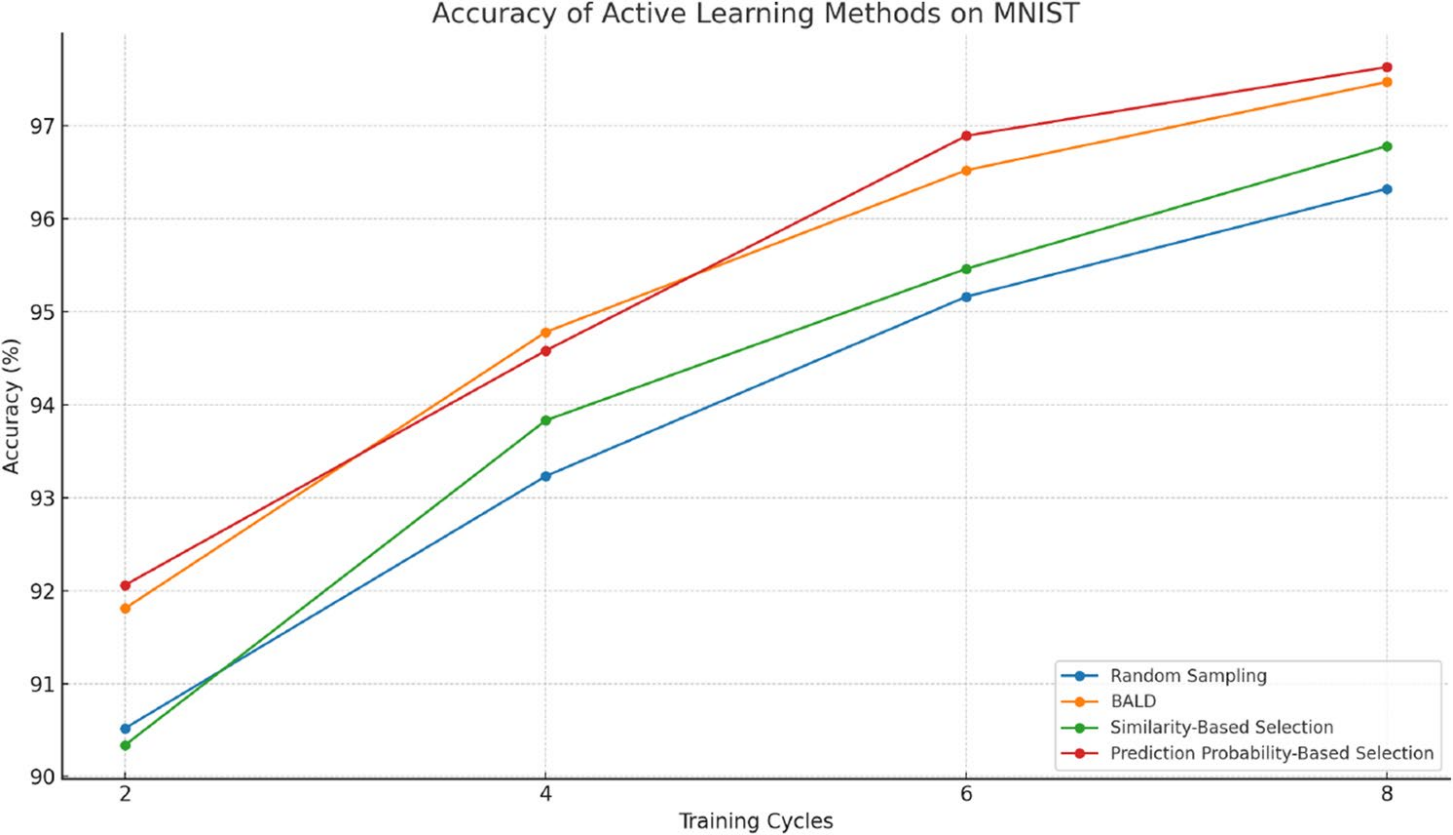
Accuracy of comparison methods on MNIST.

The experimental results on the MNIST dataset are presented in Table 5. Our methods demonstrate significant performance improvements over the cycles as illustrated in Fig. 8. Notably, the Prediction Probability-Based Selection method shows a steady increase in accuracy, achieving 97.63% in the eighth cycle, which is higher than the other methods, including Random Sampling and BALD. For Competence-Based Active Learning, the performance on MNIST and ImageNet is summarized in Tables 6 and 7, respectively. On MNIST, the method reaches an accuracy of 98.72% by the eighth cycle.

**Table 5:** Accuracy of Active Learning Methods on MNIST.

Method	Cycle 2	(%)Cycle 4	(%)Cycle 6	(%)Cycle 8
Random sampling	90.52	93.23	95.16	96.32
BALD	91.81	94.78	96.52	97.47
Similarity-based selection	90.34	93.83	95.46	96.78
Prediction probability-based selection	92.06	94.58	96.89	97.63

**Table 6:** Accuracy of competence-based active learning on MNIST.

Cycles	Cycle 2	Cycle 4	Cycle 6	Cycle 8
Number of labeled data	10,000	15,000	20,000	25,000
Competence-based active learning	93.53%	96.85%	98.27%	98.72%

**Table 7:** Accuracy of competence-based Active Learning on ImageNet.

Cycles	Cycle 2	Cycle 4	Cycle 6	Cycle 8
Number of labeled data	20,000	40,000	80,000	120,000
Competence-based active learning	55.03%	62.55%	68.37%	72.02%

#### Performance on ImageNet

**Figure 9:**
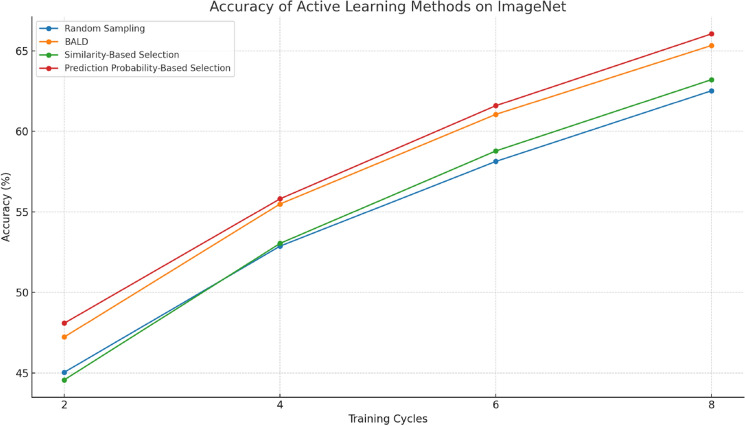
Accuracy of comparison methods on ImageNet.

Table 8 illustrates the performance of our Active Learning methods on the ImageNet dataset. Similar to the results on MNIST, our methods, particularly the Prediction Probability-Based Selection, consistently outperform Random Sampling and BALD across all cycles, as shown in Fig. 9. In the eighth cycle, this method achieves an accuracy of 66.05%, demonstrating its effectiveness in handling more complex and diverse datasets. For Competence-Based Active Learning, the performance on MNIST and ImageNet is summarized in Tables 6 and 7, respectively. On ImageNet, a more challenging dataset, the method still shows significant improvement, achieving an accuracy of 72.02% in the eighth cycle, illustrating its robustness across different types of datasets.

**Table 8:** Accuracy of active learning methods on ImageNet.

Method	Cycle 2 (%)	Cycle 4 (%)	Cycle 6 (%)	Cycle 8
Random sampling	45.04	52.87	58.13	62.52
BALD	47.23	55.49	61.05	65.33
Similarity-based selection	44.57	53.04	58.78	63.21
Prediction probability-based selection	48.09	55.81	61.59	66.05

## Discussions

Image classification plays a crucial role in various industries and sectors, but the need for large labeled datasets poses significant challenges in model development. Active Learning offers a promising solution by reducing the cost and effort of labeling data through the selection of informative samples. In this study, we propose three novel Active Learning methods, namely similarity-based selection, prediction probability-based selection, and competence-based Active Learning, to address the limitations of traditional approaches and enhance the efficiency and effectiveness of sample selection in image classification.

### Performance of proposed methods

In comparison to existing methods, our proposed Active Learning methods demonstrate several advantages. Firstly, similarity-based selection offers a distinct improvement over random sampling, especially after several rounds of training. By leveraging the similarity between unlabeled images and the labeled dataset, this method ensures that the selected unlabeled data is representative of the already labeled data, thereby reducing selection bias and promoting better coverage of the data distribution space. This aligns with the findings of Sener et al., who emphasize the importance of selecting diverse samples to enhance the robustness and generalization ability of the model. In contrast, random sampling lacks the capability to strategically select informative samples, leading to suboptimal learning efficiency, as supported by the observations of Settles.

Secondly, prediction probability-based selection consistently outperforms random sampling and even the commonly used BALD method. By incorporating the model’s current performance on the unlabeled data, this method effectively identifies samples that are both informative and within the model’s learning capacity. This is in line with the works of Yoo et al. and He et al., who utilize loss modules to learn the loss of a target model and select data based on their output loss. The effectiveness of prediction probability-based selection in reducing training time and achieving higher accuracy is further supported by the experiments conducted on CIFAR10 and CIFAR100 datasets, consistent with the findings of previous research^16,25^.

Lastly, competence-based Active Learning stands out by adapting the selection strategy to the model’s learning pace and capacity. Inspired by the concept of curriculum learning, this method progresses from easier to more challenging concepts, enabling better optimization and reducing the risk of converging to local optima. This aligns with the research of Bengio et al. and Weston et al., who highlight the importance of gradually increasing the difficulty of training samples. The effectiveness of competence-based Active Learning in reducing training time and achieving higher accuracy with fewer training rounds is demonstrated in the experiments conducted on CIFAR10 and CIFAR100 datasets.

While our proposed methods demonstrate promising results, it is crucial to acknowledge their limitations. In the case of Similarity-based Selection, we observed fluctuations in performance during CIFAR10 experiments, necessitating multiple training cycles to consistently surpass random sampling. This observation resonates with the insights of Sinha et al.^27^, who discuss the impact of a discriminator’s output quality on the effectiveness of diversity-based sample querying in an adversarial context. Additionally, the applicability of Competence-based Active Learning is limited. It may not be optimal when the model’s learning capabilities are already finely tuned or when exploring a broader range of the data space is preferable. This perspective is supported by Bengar et al., who emphasize the significance of integrating Active Learning with self-supervised pre-training for more diverse data exploration.

### Application scenarios of proposed methods

Similarity-based selection ensures that the selected unlabeled data aligns with the underlying data distribution of the labeled data, thus enhancing the model’s robustness and generalization ability. Similarity-based selection is especially beneficial in scenarios where preserving the overall data distribution is crucial, such as image classification tasks with complex and diverse datasets. In such applications, ensuring broad coverage of different geographic features and conditions is crucial, and Similarity-based Selection effectively aids in capturing a wide range of data variability, enhancing model accuracy in diverse scenarios. This aligns with the findings of Sener et al., who emphasize the importance of representative-based methods in enhancing the robustness and generalization ability of the model^8^.

By evaluating the model’s current performance on the unlabeled data, Prediction probability-based selection method effectively identifies samples that are both informative and within the model’s learning capacity. Prediction probability-based selection is well-suited for scenarios where the initial trained model exhibits relatively high accuracy. It can be particularly useful in tasks where continuous learning and adaptation are required, such as image classification in dynamic and evolving environments. This aligns with the work of Yoo et al., who employ a loss module to learn the loss of a target model and select data based on their output loss^7^. The selection based on the model’s prediction probabilities ensures that the new data incorporated into the training set is both informative and aligned with the model’s current knowledge. In such context, our method can help in efficiently updating the classification model to adapt to new product types or styles, based on the model’s current performance, thereby maintaining high accuracy in a dynamically changing inventory.

Competence-based Active Learning method adapts the selection strategy to the model’s learning pace and capacity, making it particularly suitable for deep learning models that are prone to getting stuck in local optima. A practical use case for this method is in medical image analysis, such as in the classification of radiology scans. Here, the gradual adaptation to more complex cases as the model’s competence improves can be crucial for ensuring high accuracy and reliability, particularly when dealing with a wide range of medical conditions and image qualities. This concept aligns with the ideas of curriculum learning, where models gradually tackle more challenging tasks after mastering easier ones^29^. Competence-based Active Learning reduces training time and resource requirements while achieving higher accuracy.

## Conclusions

Our research presents three innovative Active Learning methodologies tailored for training neural machine learning models in image classification tasks. Of these, the Prediction Probability-based Selection method consistently demonstrates remarkable performance on the CIFAR10 and CIFAR100 datasets. Although the Similarity-based Selection method yields only modest improvements on the CIFAR10 dataset, it still plays a significant role in enhancing overall model performance. Crucially, the Competence-based Active Learning approach outperforms other methods in achieving higher image classification accuracy for both datasets, underscoring its effectiveness and efficiency in neural model training.

Future research should focus on integrating additional image features to more effectively quantify image dissimilarities, thereby improving performance in low-resource scenarios. Expanding experiments to include larger datasets and more iterations, as well as comparing against robust, state-of-the-art baseline systems, will provide a more comprehensive evaluation of these methods. As the domain of machine image classification rapidly advances, it is imperative that data selection methodologies evolve correspondingly, ensuring alignment with the unique characteristics and demands of current models. Continual adaptation and refinement of these methods are crucial for advancing the efficacy and accuracy of image classification models.

## Data Availability

The data that support the findings of this study are openly available at http://www.cs.toronto.edu/~kriz/learning-features-2009-TR.pdf, reference number^9^.
